# Development of an Inexpensive Harnessing System Allowing Independent Gardening for Balance Training for Mobility Impaired Individuals

**DOI:** 10.3390/s21165610

**Published:** 2021-08-20

**Authors:** McPherson Newell, Ann Reinthal, Debbie Espy, Beth Ekelman

**Affiliations:** 1 School of Engineering, Mercer University, Macon, GA 31207, USA; mcpherson.hughes.newell@live.mercer.edu; 2 School of Health Sciences, Cleveland State University, Cleveland, OH 44115, USA; d.espy@csuohio.edu (D.E.); b.ekelman@csuohio.edu (B.E.)

**Keywords:** balance training, harness system, wearable sensors, rehabilitation, accelerometry, markerless motion capture

## Abstract

Balance is key to independent mobility, and poor balance leads to a risk of falling and subsequent injury that can cause self-restriction of activity for older adults. Balance and mobility can be improved through training programs, but many programs are not intensive or engaging enough to sufficiently improve balance while maintaining adherence. As an alternative to traditional balance training, harnessed gardening sessions were conducted in an urban greenhouse as an example of a community activity through which balance and mobility can be trained and/or maintained. An inexpensive multidirectional harness system was developed that can be used as an assistive or rehabilitative device in community, private, and senior center gardens to allow balance or mobility-impaired adults to participate in programming. Two wearable sensor systems were used to measure responses to the system: the Polhemus G4 system measured gardeners’ positions and center of mass relative to the base of support, and ActiGraph activity monitors measured the frequency and intensity of arm movements in garden as compared to home environments. The harnessed gardening system provides a safe environment for intense movement activity and can be used as a rehabilitation device along with wearable sensor systems to monitor ongoing changes.

## 1. Introduction

Independent mobility is key to participating in numerous life activities [1] and adequate balance is a requirement for mobility [1]. Poor balance and its associated fall risk increase with aging and disability, resulting in one of every three older adults falling yearly, sometimes with resultant injuries [2]. In many cases, falling leads to a fear of falling and subsequent self-limiting of activity [3,4]; in a downward spiral, this inactivity leads to further impairments in balance as well as other areas such as strength, motor control, and cardiorespiratory fitness [5]. Community and individualized mobility and balance training programs are frequently used to break this cycle. However, many balance-impaired individuals do not complete effective interventions [6]. There are several reasons why current balance and mobility training is not always effective. First, typical training programs involve proactive, or self-initiated, movement practice, which is often not completed at an intense enough dosage to elicit a change [7,8] and does not train reactive responsiveness to unexpected falls [9]. Another explanation is that training requires substantial amounts of practice which must be maintained over time by continued practice [10]. Compliance with initial and continued practice is problematic. Finally, clinic-based activities may not be similar enough to real life, lacking the necessary task and environmental complexity to generalize outside of the clinic [11].

To address these issues, we have adapted an inexpensive multidirectional harness system (MDHS) that allows balance impaired individuals to garden independently. We believe there are two types of users for the MDHS. First are those who use the system as an assistive device, thereby maintaining their current function by remaining more active, breaking the cycle of increasing inactivity due to limited mobility and balance. Second are the individuals who use the system as a rehabilitation device and improve their mobility due to the use of this system, perhaps learning to function without the device. The focus of this article will be using the MDHS for rehabilitation.

We hypothesize that there are three critical elements in the use of this technology: the MDHS, the natural community setting, and the increased activity level. The safety imparted by the MDHS allows more risk-taking by the harnessed wearer, providing a higher intensity and therefore more effective practice than is afforded by a non-harnessed environment where falls are possible. Gardening, as an activity, requires significant amounts of reaching and bending as well as movement over irregular surfaces, with the MDHS allowing safe exploration of these balance challenges. In addition, MDHS training can be easily individualized to people at multiple levels of function, again allowing practice intensity to be maintained. Completing training in the garden, a community setting, rather than a clinic, can result in better compliance with training. Better compliance, in turn, provides the necessary initial mass practice as well as supporting continued ongoing practice. In addition to being a social community activity, gardening has been shown to have therapeutic benefits [12,13]. The garden’s complex, real-life environment allows for more effective generalization of improvements in balance beyond the single therapeutic activity/setting. Finally, using the harness breaks the cycle of increasingly sedentary activity choices for the user, increasing overall movement and steps taken over the course of the day.

The purpose of this study is twofold. First, it will describe the development and implementation of the harnessed mobility system using commercially available components along with the wearable sensor system required to quantify change. Second, it will examine initial feasibility issues for individuals interested in gardening but unable to do so without assistance [14,15]. Specific feasibility considerations addressed include acceptability (satisfaction, intent to continue use, and perceived appropriateness), practicality (positive and negative effects on participants, ability of participants to carry out the activity, and basic cost analysis), and limited efficacy testing to determine intended effects of the intervention.

## 2. Materials and Methods

### 2.1. System Design and Development

This section describes the design and development, over three gardening seasons, of the MDHS, its associated components, and the wearable sensor systems and is summarized in Table 1. The project was approved by the Cleveland State University Institutional Review Board (IRB-FY2018-71). 

**Figure 1:**
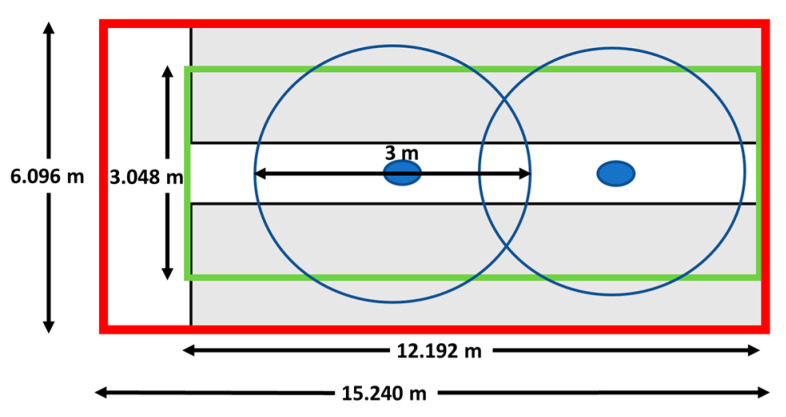
Garden dimensions: gardening area in gray; MDHS coverage green inner rectangle; and Polhemus sensor coverage two overlapping blue circles.

The harness framework was retrofitted into a 50′ × 20′ community urban high tunnel greenhouse that included a forty-foot length of the actual garden. Our goal was to allow harnessed gardening in as much of this area as possible. The 40′ × 10′ harness framework (two rails connected by a transit) is diagramed in Figure 1 and was designed by MASSREHAB (Clayton, OH, USA) to fit within the covered garden. Because we were retrofitting an area with height limitations, the system could not accommodate work along the two outer edges of the garden, although some reaching could occur outside of the ten-foot width. The gardener wore a harness connected via a spreader bar, ropes, and carabiners to the MDHS framework. A variety of different commercially available fall arrest harnesses were used with this system depending on user need.

**Figure 2:**
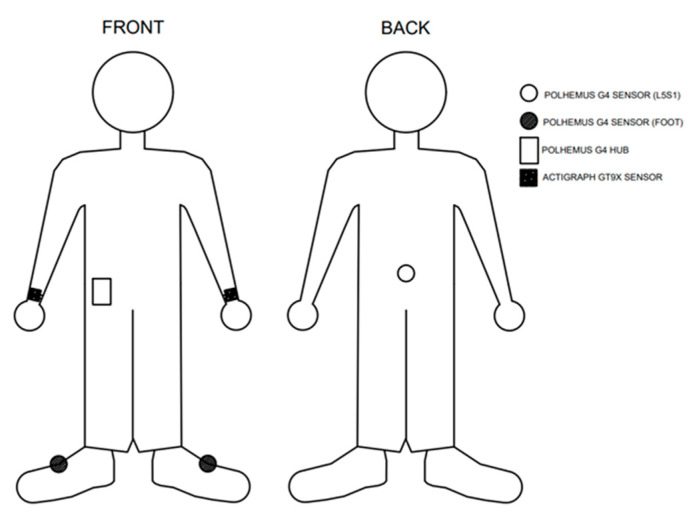
A diagram of the sensor locations on the participant’s body. The Polhemus G4 sensors (circles) are attached to each shoe and to the participant’s back, the Polhemus G4 hub is worn on the waistband, and the ActiGraph GT9X sensors (squares) are worn on the wrists.

We wanted a low-profile and easily donned wearable sensor system that could monitor the position of the gardener (standing, walking, sitting, kneeling, transitioning between positions) as well as analyze the movement of the center of mass (COM) relative to the base of support (BOS). The Polhemus A/C electromagnetic G4 (Colchester, VT, USA) sensor system was chosen; it provided an absolute 0 origin allowing the desired kinematic analysis with only three markers as compared to inertial sensor systems with relative origins that require a much larger number of sensors. One marker was placed between the second and third metatarsal bases of each foot and the third marker was placed on the lower back at L5-S1 of the spine as illustrated in Figure 2.

In addition, we wanted to do activity monitoring. While the Polhemus system provides step and position data, we used GT9X Link Activity Monitors (ActiGraph; Pensacola, FL, USA), containing a 3-axis accelerometer, to measure upper extremity activity in the garden.

An inexpensive active assist strap system was developed, consisting of elastic bands of varying densities (sized to assist gardeners of differing weights) connected to carabiners on the back of each harness thigh strap and to the top harness connection. The elastic band tightens when the user sits or kneels, and the subsequent spring release provides assistance to initiate standing, which is typically the most difficult part of this motion. 

The one-handed harness height adjuster (MASSREHAB, Clayton, OH, USA) was needed when gardeners wished to move between standing and kneeling or sitting. It is comprised of pulleys, a locking mechanism, carabiners, and rope. It allows gardeners to unlock the mechanism with one hand at or below shoulder level by flicking the free length of rope; they next adjust the amount of slack in the rope to change positions and then relock the mechanism in the new position to prevent falls. For example, when transitioning from standing to sitting, the user unlocks the locking mechanism, sits down, and re-locks the height adjuster.

### 2.2. System User Evaluation 

One woman with multiple sclerosis (P1) and two adults with chronic stroke (one female—P2, one male—P3) participated in this initial system development and feasibility study. Individuals with a range of functional mobility levels were purposefully selected to participate in the initial group. P1 used a power wheelchair and only ambulated short distances with a wheeled walker, P2 ambulated independently with a quad cane, and P3 did not use an assistive device when walking. They gardened using the harness system for eight to twelve sessions, each about one hour in length.

Both quantitative and qualitative methods of analysis were used to evaluate the use of the harness system. The qualitative data consisted of thematic analysis of interviews conducted with the study participants after the completion of the gardening intervention. Quantitative pre-post measurement included clinical measures of strength, balance, mobility, and participation. These data are presented here only in reference to specific feasibility questions and include pre-post test data for two clinical tests. The Timed Up and Go (TUG) measures the time it takes to stand up, walk 3 m, turn, return, and sit down; it indicates mobility level and predicts fall risk [16]. The Berg Balance Scale (BBS) is a 14-item exam of a series of static and dynamic tasks that require balance; it also assesses fall risk [17].

Ongoing measurement using wearable sensor systems included upper extremity activity monitoring and gardening position and position change detection (walking vs. standing, sitting, or kneeling) as measures of activity levels and analysis of the movement of the center of mass (COM) relative to the base of support (BOS) [1].

### 2.3. Data Analysis

The G4 data is exported to MATLAB (Mathworks; Natick, MA, USA) and processed using custom-written software. Limb length and distance from the foot marker to the front, back, and sides of the foot are input into the code for each user. Relative marker positions allow the determination of single/double limb support in standing/walking as well as sitting and kneeling positions.

The participant’s BOS is calculated based on the position at any given point in time. The L5-S1 sensor approximates the participant’s COM [18]. The code calculated and displayed the BOS as well as the center of the BOS which forms a varying irregular polygon depending on the gardener’s position. On this was superimposed the moving COM, displaying how close the COM was from the edge of the BOS. This analysis then quantifies the distance of the COM from the edge of the BOS and assesses the velocity of COM movement within and outside of the BOS [19].

GT9X Link Activity Monitors (ActiGraph; Pensacola, FL, USA), measured upper extremity activity in the garden as compared to the home environment. While inertial sensor-based step counters have been widely used to quantify lower extremity activity in walking, activity trees provided a means of quantifying and visualizing arm activity [20,21]. From the accelerometer data, a density plot is derived that quantifies the intensity of bilateral upper extremity motion. One activity monitor was worn on each wrist during the garden sessions as well as during waking hours at home. The accelerometers in the ActiGraph inertial sensors record the acceleration of the participant’s arm in three axes (x, y, and z) as well as the vector magnitude of the acceleration. Data analysis includes the creation of activity trees, showing the overall distribution of activity of each arm. These activity trees are composed of bivariate histogram density plots graphing the magnitude ratio (1,2) versus the bilateral magnitude of acceleration, where the magnitude ratio is:

(1) $$\ln\left(\frac{\mathrm{vector}\;\mathrm{magnitude}\;\mathrm{of}\;\mathrm{paretic}\;\mathrm{arm}}{\mathrm{vector}\;\mathrm{magnitude}\;\mathrm{of}\;\mathrm{non}-\mathrm{paretic}\;\mathrm{arm}}\right),$$

which is in this case:

(2) $$\ln\left(\frac{\mathrm{right}\;\mathrm{vector}\;\mathrm{magnitude}}{\mathrm{left}\;\mathrm{vector}\;\mathrm{magnitude}}\right).$$

where the bilateral magnitude is the summation of the vector magnitudes of acceleration for both arms [18]. The bilateral magnitude indicates the intensity of movement, with a larger bilateral magnitude corresponding to a more strenuous or intense movement, and the magnitude ratio reflects the comparative usage of each arm. A negative magnitude ratio indicates that one arm was used more than the other, and a positive magnitude ratio indicates the opposite, with values further from zero indicating a greater discrepancy between arms. A magnitude ratio of zero indicates that both arms were used the same amount. The density plot component of the activity trees represents the total duration of movement performed by the participant by reflecting the frequency at which that value occurs, where warmer colors represent longer duration, with dark red being the longest, and cooler colors represent shorter durations, with dark blue being the shortest. 

Activity trees for persons without arm impairment are fairly symmetrical, with the greatest values of bilateral magnitude corresponding to a magnitude ratio of zero, and the longest durations of movement also occurring with bilateral use [20,21] since most arm movements are bilateral [21]. However, activity trees for individuals with hemiplegia post-stroke or other unilateral arm impairment are asymmetrical, reflecting greater use of the non-paretic or more normal arm, and are smaller than those of individuals without unilateral arm impairment, representing both lower intensity and duration of movement overall [20,21]. In addition, upper extremity activity trees reflect the overall level of arm use over time, similar to a step counter monitoring leg use in walking.

## 3. Results

### 3.1. Development and Implementation

The harness system was developed gradually over the course of three gardening seasons, with new components added annually as illustrated in Table 1. The active assist band was needed and used with two of the gardeners. The one-handed height adjuster was initially trialed by P2 and used regularly by P3. Only sensor data from the final configuration used in Year 3 is presented.

### 3.2. Acceptability, Practicality, and Limited Efficacy Testing

Qualitative interviewing indicated that P1 and P2 felt the harness system was helpful while P3 found the system constrictive; however, the clinician investigators did not feel P3 would have been safe gardening as actively as he chose to do without wearing the harness or being carefully guarded to prevent falls. P1 and P2 would have continued gardening using the harness system after the conclusion of the data collection period if it were available. In all cases, the harness system prevented falling during various gardening activities and allowed the participants to do more in the garden than they could have safely performed without hands-on guarding by a clinician. In the case of P1, this was reaching activities in sitting and more transitions between sitting and standing using the active assist straps, while P2 was able to bend and reach with one leg up on the raised bed. P3 bent, reached, and freely stepped in and out of the raised beds. 

TUG and BBS scores pre- and post-gardening are presented in Table 2. BBS scores remained unchanged while TUG scores improved in two of the three participants.

The COM/BOS position measurement system went through several iterations, including the use of foot pressure sensors before the decision to use only the three G4 sensors. It took less than five minutes to don, although additional straps were added to secure the wires extending up the legs from the sensors to the waistband control unit (see Figure 2). Also, the low back sensor was attached to the skin using medical tape rather than the waistband of the participant’s trousers to decrease the excess movement that occurred when attached to clothing rather than directly to the skin. A slap wrist band was added to the activity monitors to allow independent donning at home, as this design was the easiest for P3 to attach to his non-paretic arm using his paretic arm.

### 3.3. Cost Analysis

Table 3 summarizes the cost for each component of the system.

### 3.4. Position and Movement of COM Relative to BOS Analysis

The Polhemus G4 wearable sensor system was able to accurately measure the gardeners’ positions for assessment of activity level. Specifically, the sensor data measured if the gardener was standing, walking, kneeling, sitting, or moving between these positions at any given point in time. For example, during the 60 min of Session 4, the analysis showed that P3 stood and/or walked throughout the session except for two sitting periods with a duration of less than 5 min each.

**Figure 3:**
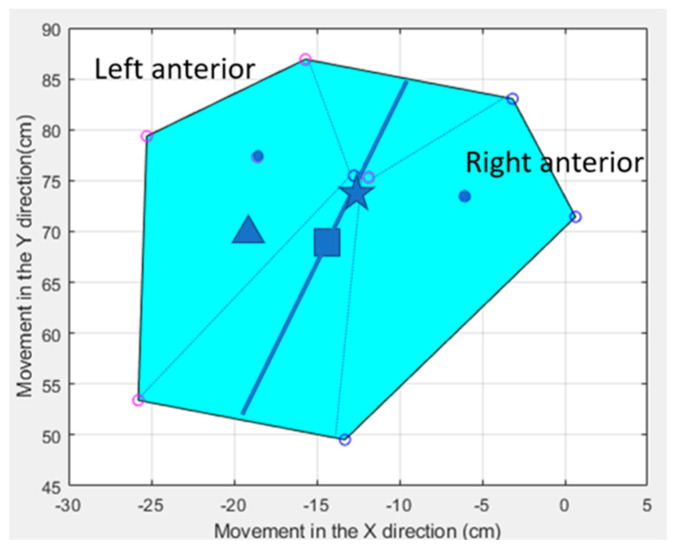
This figure shows two superimposed points in time where P3′s feet have not moved but he has moved his COM over his BOS. The BOS is the shaded area formed by the two feet. The sensor position is the solid circle on each foot; the open circles represent the edges of each foot based on measurements inputted into the code relative to the sensor position. The square is the center of the irregular polygon as calculated by the code. The star is COM 1, and the triangle is COM 2.

The code calculated and displayed the BOS and the center of the BOS. On this was superimposed the COM, calculating how close the COM was from the edge of the BOS at any given point in time, or if the COM traveled outside of the BOS as when taking a step. Figure 3 displays two timepoints, COM 1 and COM 2, when P3 is standing and shifting his weight over his feet. 

### 3.5. ActiGraph

Activity trees were generated for each of P3′s seven gardening sessions in which data was recorded. P3 also wore the ActiGraph wrist sensors at home for multiple waking hours over the course of four days (two days at a time on two occasions). The two most active independent hours for each day were determined by calculating the sum of the bilateral magnitude for each hour, and activity trees were generated for these eight hours. Because only the most active times were chosen, these did not include any waking times of minimal activity such as when watching television.

**Figure 4:**
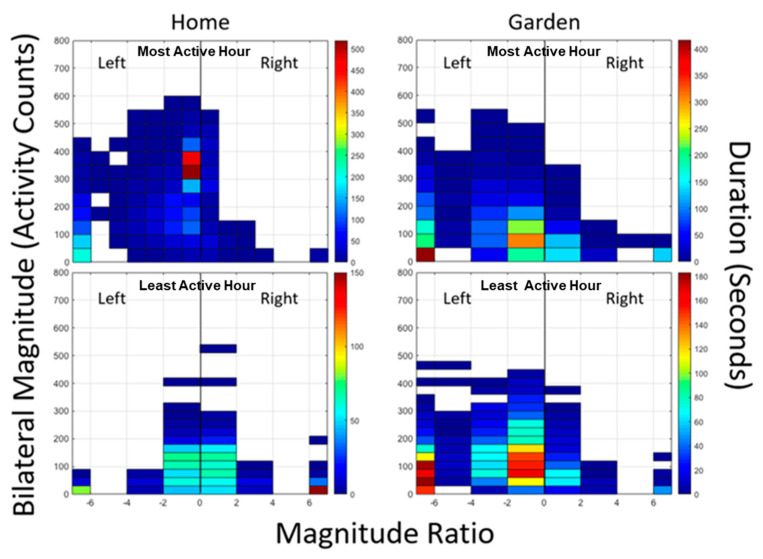
Activity trees for the most (**top**) and least (**bottom**) active measured hours from the home (**left**) and garden (**right**). The color scale indicates the duration of activity, with the reddest being the longest duration in seconds and the bluest being the shortest. Duration is scaled to the maximum for each hour.

Figure 4 shows the activity trees for the most and the least active hours at home (from only most eight active hours) and in the garden. Since P3 has right-sided hemiplegia, there is relatively less right-sided activity on all trees. It can be noted that activity in the garden as compared to the home is similar for the high activity period and somewhat higher for the low activity period.

**Figure 5:**
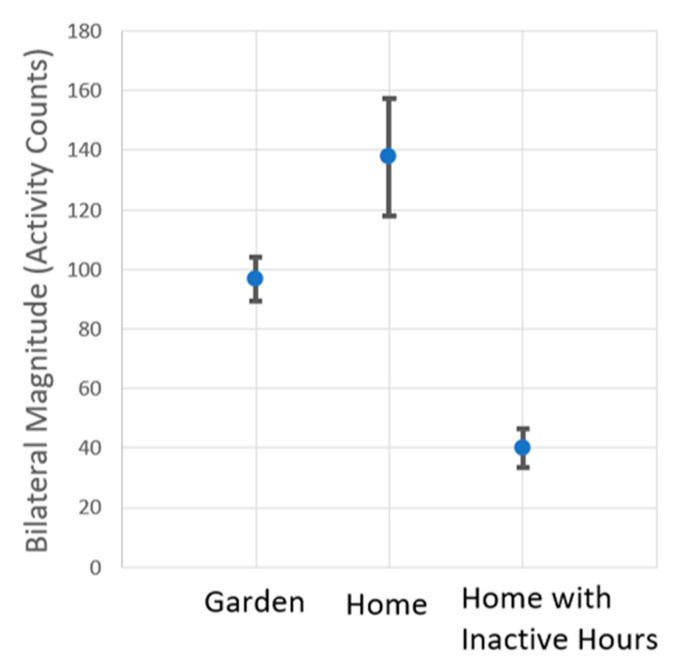
A visual t-test comparing the median bilateral magnitude for activity in the garden (Garden), most active hours of the home (Home), and total waking hours the sensors were worn in the home (Home with inactive hours). The mean of the median bilateral magnitude was 96.7 activity counts for the garden sessions, 137.7 activity counts for the most active hours of the home, and 40.0 for the total hours at home. The standard error was 8.1 for the garden, 19.7 for the most active hours at home, and 6.6 for the total measured hours at home.

A visual *t*-test (Figure 5) shows the intensity of activity in the different settings. The median bilateral magnitude, excluding periods of zero activity, was calculated for each gardening session (Garden), each of the eight hours of maximum activity from the home environment (Home), and all waking hours when at home (Home with inactive hours). Figure 5 displays the mean and standard error of the median bilateral magnitudes for the garden and two home environments; it was highest in the active hours at home and lowest during all waking hours at home. However, the activity level in the garden as compared to the active hours at home was more consistent; the median bilateral magnitude often differed highly between the most active and second most active hours at home, leading to the higher standard error for the active hours at the home environment as compared to the garden for the garden.

## 4. Discussion

The MDHS and wearable sensor systems have been trialed, and initial component development is largely complete. The harnessed gardening system was successfully used by three mobility impaired individuals with varying levels of dysfunction, from an individual using a power wheelchair and walking minimally to someone who walks independently (BBS scores from 13/56 to 56/56), showing that this system is scalable to meet varying levels of mobility impairment. 

We were able to effectively use a low-profile, three-sensor system to assess ongoing position information of the gardener (standing, walking, sitting, or kneeling) as well as analyze the COM position and motion relative to the BOS, with the exception of incorporating arm weight-bearing during gardening. While inertial sensor systems are also low profile, they lack an absolute 0 origin and therefore require many more sensors to provide similar kinematic data. Although work is ongoing to resolve this problem [22], there is currently no 3-sensor inertial sensor system that can provide this project’s desired kinematic analysis. The commercially available inertial sensor systems are also more expensive. For example, the seven inertial sensor Moveo Explorer system from ADPM is USD 17,000 as compared to the USD 8000 three-sensor Polhemus used here.

As a custom-designed project, this system, including the wearable sensors, was built for under USD 20,000 (see Table 3), which is relatively inexpensive compared to most clinical harness systems. For example, the Bioness Vector track system costs over USD 200,000. Our long-term goal is to develop an inexpensive MDHS since more expensive clinical systems are not accessible to large parts of our population, especially in a community setting. This is a critical need as our population both ages and lives with higher levels of physical disability. We believe similar systems could be installed and used in multiple community locations if costs were not prohibitive. For example, the MDHS could be used by mobility-impaired individuals in work and classroom environments; we have consulted on a system now installed in a classroom for disabled individuals allowing increased daily activity while preventing falls. The system could be installed in community, private, senior center, and school gardens as well as recreational and senior centers where it could support various types of activities. For example, we are currently designing a project in a skilled nursing facility using MDHS in resident rooms to allow activities of daily living such as walking and self-care (washing up, brushing teeth, etc.) in standing and in an activity area allowing group classes such as video gaming leagues, line dancing, and Tai Chi. While the use of an MDHS is limited to the area of the overhead gantry, we contend that this is more accessible to a larger range of individuals, especially older adults, than the exoskeleton systems currently under development due to both cost and ease of use. Harness systems also typically require the user to be more active than exoskeletons. 

Activity monitors are inexpensive and are easy to use. The activity tree analysis was able to quantify upper extremity activity levels. Since a goal of gardening is to provide activity, especially for a less used arm as is common with hemiplegia post-stroke, activity trees provide a meaningful means of assessment. With the addition of well-established pedometry [23] for step counting, activity monitoring for both the upper and lower extremities could be completed with relative ease.

Participants found harnessed gardening to be an engaging activity, with P1 and P2 both asking to continue gardening past the end of scheduled sessions. This does not typically occur in a clinical setting. Being involved in an enjoyable community-based activity is more likely to maintain compliance with physical activity than typical home exercise programs. Thus, we hypothesize that the harnessed gardening intervention, as well as other applications of the MDHS that support active mobility, will increase the frequency and duration of the movement. This, in turn, will help address the downward spiral of inactivity that occurs with mobility impairments. Thus, the MDHS has the potential to provide an effective training intervention. While the intent of this paper was to present the development and initial implementation of the MDHS with a wearable sensor system, we believe the sensor system has the ability to successfully monitor changes in activity levels. 

With further work ongoing in our lab, we hope to be able to use the analysis of the movement of the COM relative to the BOS to further explore this aspect of balance control [19]. Does use of the MDHS result in a gardener engaging in more risky and challenging behavior as demonstrated by moving the COM more quickly and/or further to the edges of the BOS as compared to a non-harnessed gardener? Rehabilitation with the MDHS may assist in addressing the motor learning transfer problem [11] by training in a more context-normal environment than the typical clinical setting. In addition, for individuals with hemiplegia, such as P2 and P3, encouraging paretic arm use in an engaging activity may increase their confidence and capability to perform bilateral movements at home as well as in the garden. A longer and larger trial with a control group will be required to begin answering these questions.

This study has several limitations. As with any newly developed mobility system, additional testing is necessary with a wider range of users. Moreover, it is unknown what dosage of gardening is needed in the MDHS in order to make clinically significant changes in balance and mobility; the limited number of sessions completed by participants in this study was not expected to result in large changes in clinical testing scores. While there was an improvement in two of the participant’s TUG scores, their BBS did not change, although the known BBS ceiling effect was evident in both P2 and P3 [18]. Finally, pedometry, as well as additional hand pressure sensors, need to be added. With additional hand sensor information added to the position coding, the sensor system will be able to assess when the individual is using one or both arms for weight-bearing. For example, a gardener often kneels and balances on one arm while reaching with the other, increasing the BOS significantly. This will allow a more accurate assessment of the movement of the COM relative to the BOS.

## 5. Conclusions

We have successfully developed an inexpensive multidirectional harness system that allows individuals with balance and mobility impairments to garden independently. The gardening system provides a safe environment for intense movement activity and can be used as a rehabilitation device. It includes wearable sensor systems monitoring ongoing changes in gardener position as well as COM relative to BOS and ongoing overall upper extremity activity.

## Figures and Tables

**Table 1:** Summary of development of MDHS with wearable sensors.

Year	Subject	Activity
1	P1	Install MDHS, develop active assist straps, develop analysis for Polhemus system
2	P2	Trial Polhemus system 1st iteration, begin trial height adjuster
3	P3	Develop analysis/trial activity monitors, trial Polhemus system 2nd iteration

**Table 2:** Pre- and post-clinical measures.

Measure	P1		P2		P3	
	Pre	Post	Pre	Post	Pre	Post
**BBS**	13/56	13/56	54/56	54/56	56/56	56/56
**TUG (s)**	56.5	59.3	23	16.9	12.4	8.8

**Table 3:** Cost of system components.

Component of System	Cost	
Multidirectional harness system	$8000	
Spreader bar, ropes, and carabiners	$400	
Harnesses	$100–$300 range each	
Polhemus G4 system with 2 hubs	$8000	
GT9X Actigraph activity monitors	$300 each	
Active assist straps–multiple sizes	$100/set	
Harness height adjuster	$700	
